# Identification of Two Distinct Stem Cell Clusters, Lrig1-Derived and Wnt/CD44-Dependent, in Corneal Epithelium

**DOI:** 10.3390/ijms26136383

**Published:** 2025-07-02

**Authors:** Laurent Barnes, Evangelia Konstantinou, Jean-Hilaire Saurat, Alexandre Moulin, Gürkan Kaya

**Affiliations:** 1 Department of Dermatology, University Hospital of Geneva, CH-1205 Geneva, Switzerland; laurent.barnes@cutiss.swiss; 2 Department of Medicine, University of Geneva, CH-1206 Geneva, Switzerland; evangelia.konstantinou@unige.ch; 3 Department of Clinical Pharmacology and Toxicology, University of Geneva, CH-1206 Geneva, Switzerland; jean.saurat@unige.ch; 4 Jules-Gonin Eye Hospital, Lausanne University, CH-1004 Lausanne, Switzerland; alexandre.moulin@fa2.ch; 5 Department of Clinical Pathology, University Hospital of Geneva, CH-1205 Geneva, Switzerland

**Keywords:** Lrig1, CD44, Wnt, cornea, tamoxifen, ablation, stem cell

## Abstract

We previously showed that selective suppression of CD44 in the corneal epithelium leads to structural abnormalities in the mouse cornea. Our comparative studies of young and aged ocular biopsies revealed that CD44 expression is downregulated in aged corneas, while leucine-rich repeats and immunoglobulin-like domain 1 (Lrig1+) stem cells remain preserved in the peripheral limbus. These findings suggest an age-related shift in the corneal stem cell compartmentalization, characterized by impaired CD44 expression in the central cornea and preservation of Lrig1+ stem cells in the limbus, which become the main stem cells in the senescent cornea. To investigate this further, we performed topical tamoxifen-inducible, diphtheria toxin-mediated ablation of Lrig1+ stem cells in mouse corneas. We then assessed both activated and non-activated beta-catenin expression in wild-type (WT) and CD44 knockout (CD44KO) mice, given that CD44 modulates the Wingless-related integration site (Wnt) pathway. Our results indicate that two distinct stem cell populations operate in the mouse cornea: Lrig1-derived stem cells and Wnt-activity/CD44-dependent stem cells. The Lrig1-derived cells act as a reservoir of quiescent stem cells that regenerate the cornea upon injury, whereas under homeostatic conditions, the Wnt-activity/CD44-dependent stem cells are primarily responsible for corneal renewal. In the aged cornea, the loss of CD44 expression leads to reduced Wnt signaling, making the tissue increasingly dependent on Lrig1+ stem cells for regeneration. In mice, Lrig1+ stem cells are capable of sustaining permanent corneal renewal, even in the absence of CD44.

## 1. Introduction

We have shown that selective in vivo suppression of CD44 in keratinocytes resulted in abnormalities in the corneal epithelium in mice and in a skin phenotype reminiscent of dermatoporosis [1,2]. We explored the heterogeneity of epidermal stem cells located in the basal layer of human epidermis and whether this heterogeneity was modified during aging [3]. According to our data, two types of stem cells, the Epidermal Growth Factor Receptor (EGFR)+CD44+ and the EGFR inhibitor Lrig1+ (EGFR-CD44-) stem cells, showed important changes in the aged epidermis. Located in the basal layer of epidermis, the EGFR+CD44+ cells play an important role in skin biology by maintaining skin homeostasis, contributing to wound healing and in the maintenance of stem cells. In contrast, the Lrig1+ cells, located in the interfollicular and follicular zones in human skin, would rather be activated in wounding of the epithelium. In case of injury, they maintain the skin homeostasis and prevent the excessive proliferation of cells and as a result the tumorigenesis [4,5,6]. The Lrig1+ stem cells were shown to be the only ones able to renew all compartments of the epidermis (interfollicular epidermis, sebaceous gland and the hair follicle) [7,8,9]. In homeostatic conditions, the Lrig1+ stem cells remain quiescent, and are believed to have more stemness properties than other epidermal cells located in the basal layer [10].

In addition, our data also underlined that in senescent human atrophic epidermis, the EGFR+CD44+ cells tend to disappear together with a loss of Wnt signaling activity, while the Lrig1+ stem cells are preserved. The hallmark of aged epidermis is characterized by a reduced expression of CD44, reduced Wnt signaling and a cluster of Lrig1+ stem cells left as the last cluster of stem cells for renewing the epidermis. We have also confirmed this phenotype in a CD44KO murine model of senescent human atrophic epidermis.

Given the structural and functional similarities between epidermis and the corneal epithelium, as both of them are stratified and self-renewing epithelial tissues, we hypothesized that a similar compartmentalization may also occur in cornea. In more detail, the cornea is composed of proliferating epithelial cells in the basal layer and differentiating to form vertical rows of cells stacked together. In the eye, some stem cells have been identified such as Lrig1+ cells which are present in retina and they are characterized as retinal progenitor cells with regenerative potential [5,11,12]. They can proliferate and differentiate into various retinal cell types. Moreover, Lrig1+ cells are present in the corneal epithelium where they are involved in the maintenance and regeneration of the corneal epithelium [11]. They play an important role in maintaining the integrity and function of the corneal surface. Lrig1+ cells are also found in the lens, where they are probably involved in local tissue maintenance and repair. Furthermore, as in other tissues, these cells protect against excessive tissue proliferation and prevent the growth of tumors [5,11]. Apart from Lrig1+, other types of stem cells are also present in the eye such as limbal epithelial stem cells (LESCs), which are important for regeneration of corneal surface or corneal stromal stem cells (CSSCs) that contribute to the regeneration of corneal stroma [13,14].

Recent studies have identified markers that are expressed in stem cells in the corneal epithelium, such as p63, ATP-binding cassette sub-family G member 2 (ABCG2), cytokeratin 15 and Notch signaling-related markers [15,16]. Li et al. found that the cells which express these markers are localized in the basal layer of the corneal epithelium in a murine model [17]. However, there are no markers that are exclusively expressed in corneal stem cells and further studies are required to identify potential markers specific for the corneal epithelium.

As the cornea surface is exposed to sunlight, the regulation of the corneal homeostsasis can be expected to be partially regulated similarly to the epidermal homeostasis. Description of slow-cycling epithelial basal cells in the limbus of the mouse cornea suggested that the limbus is the niche for the corneal stem cells [17]. However, this observation was challenged by the presence of oligopotent stem cells, such as corneal limbal stem cells and conjunctival stem cells, on the entire ocular surface, including the cornea [18].

As mentioned above, the presence of different stem cell populations in the corneal epithelium is already known, but the specific roles of Lrig1+ cells and CD44-dependent stem cells and their role during aging remains poorly understood. It is also unclear whether there is a decline in the expression levels of CD44 and to what extent Lrig1+ stem cells can compensate for this loss. With the hypothesis that corneal renewal is partially similar to skin renewal, we demonstrate, in the current study, a downregulation of CD44 in the center of aged cornea with a reduction in Wnt pathway activity. Our results also highlight an increased activity of Lrg1+ stem cells in aged cornea. The findings of our study are significant because they reveal that Lrig1+ stem cells may serve as a regenerative reservoir during aging or in CD44-deficient tissues.

## 2. Results

To identify if the presence of CD44 and Lrig1+ cells is altered during age, young and aged ocular biopsies were used. These results demonstrated in aged cornea a downregulation of CD44 at the center of the cornea with an increased peripheral and paracentral expression of Lrig1.

**Figure 1:**
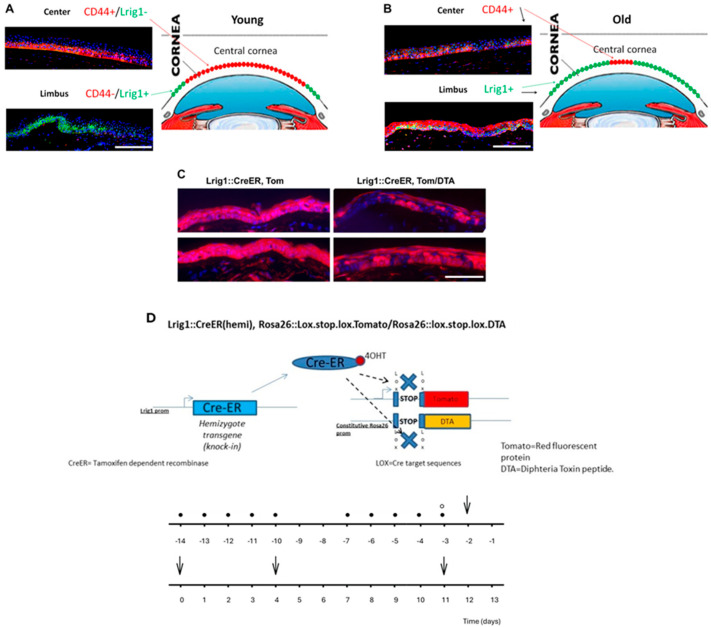
(**A**) In young human cornea, CD44 is highly expressed in the basal layer of the central cornea (red fluorescence) (48 ± 5 cells) and Lrig1 in the limbus (green fluorescence) (43 ± 5 cells). (**B**) In old human cornea, CD44 expression is decreased in the center (25 ± 5 cells, *p* = 0.005, Student’s *t* test) and Lrig1-positive cells extend from limbus to the central cornea (30 cells, original magnification). The quantification of CD44+ and Lrig1+ cells for each group was performed at 3 high magnification fields per subject. (**C**) Topical tamoxifen inducible diphtheria toxin (DTA)-mediated ablation of Lrig1+ stem cells in mouse cornea. Tomato red fluorescent protein (Tom) fluorescence in red 2 weeks after the ablation. Control = 100% of cells are red (Lrig1-derived cells) (left panel). Control = 50% of cells are red (Lrig1-derived cells), and 50% are non-red (right panel). (**D**) Transgene construct and the protocol of Lrig1 in vivo ablation and cell lineage tracing (• 4-OHT topical application; ° tamoxifen injection; ↓ cornea sample analysis). Scale bars = 87 µm.

We then performed Lrig1+ ablation in transgenic mice in order to investigate the origin of cells that are present in the cornea and are able to renew it (Figure 1).

**Figure 2:**
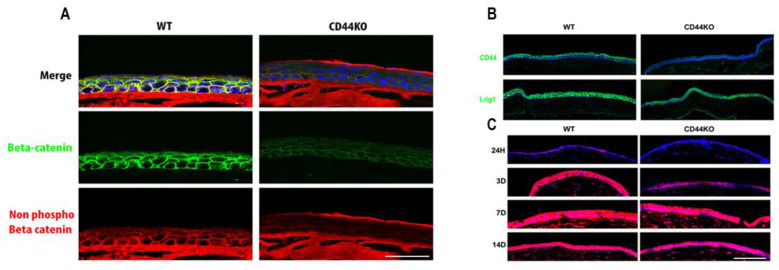
(**A**) Beta-catenin (green fluorescence) and non-phospho beta-catenin (red fluorescence) staining in the cornea of WT versus CD44KO mice. Wnt pathway seems to be downregulated in the absence of CD44. (**B**) Lrig1 (green fluorescence) is still expressed in CD44KO mouse cornea (upper panel). (**C**) Cell lineage tracing experiments in WT versus CD44KO mice. Red = Tomato was activated in Lrig1+ cells upon tamoxifen treatment (lower panel). Note that 100% of cells are red (Lrig1-derived cells) in WT animals already after 3 days, however in CD44KO animals only 70% of cells are red (Lrig1-derived cells) after 3 days, and 100% red after 7 days (Lrig1-derived compartment is in blue). No difference was observed after 14 days. WT and CD44KO transgenic mice expressing an inducible form of the Cre recombinase under the control of the Lrig1 promoter and a Cre reporter transgene coding for the tomato fluorescent protein were injected twice with 2 mg of tamoxifen in the first 24 h in order to induce the recombinase activity and to remove the codon stop that prevents the constitutive expression of the tomato protein in Lrig1 expressing corneal epithelial cells. Animals were killed at each timepoint after the tamoxifen injections. Eyes were fixed overnight in formalin and then included in Tissue-Tek OCT compound, Sakura Finetek Europe Alphen aan den Rijn, The Netherlands, and stored frozen. Cornea sections were further prepared with a cryostat (−20 °C), dried, and mounted on histological slides with the DAPI fluoromount -G^(c)^ (SouthernBiotech, Birmingham, AL, USA). Scale bars = 87 µm.

To further examine if the decline in CD44 expression during aging has an effect in the expression of Wnt signaling pathway, samples from WT and CD44KO mice were used and the expression of the activated form of beta-catenin was detected. Moreover, cell lineage tracing experiments were performed to detect if the Lrig1+ cells are present in CD44KO mice (Figure 2).

## 3. Discussion

Our preliminary studies in a group of young versus aged ocular biopsies showed that CD44 expression is downregulated in aged cornea, while the Lrig1+ stem cells are preserved in the peripheral limbus and extended to the central cornea. The modifications of the cornea stem cell compartmentalization in aged cornea seem to implicate an impaired expression of CD44 in the center of the cornea, as well as an expansion of Lrig1+ stem cells from the limbus to the corneal center, which are becoming the predominant stem cell population responsible for corneal renewal in the aged tissue (Figure 1).

To further explore the functional roles of these populations, we performed ablation experiments of Lrig1+ cells in mice. We observed that following the ablation of Lrig1+ stem cells in the cornea, there is a mixture of Tomato+ and Tomato- cells. This observation leads to the conclusion that a part of the cornea is composed of Lrig1-derived cells (cells deriving from Lrig1+ cells having survived the ablation, Tomato+ cells) and of non-Lrig1-derived cells (Tomato- cells). In contrast, in the control mice, all cornea cells appeared to be Lrig1-derived as all were Tomato+ (Figure 1). These findings suggest that two kinds of stem cells seem to be able to renew the cornea: the Lrig1+ cells and Lrig1-, but probably also EGFR+CD44+ cells.

Expanding upon a previous study that demonstrated the role of CD44 in modulating the Wnt signaling pathway, our current results show that the activity of the Wnt pathway is reduced in the corneas of CD44KO mice [3]. However, the corneal homeostasis is maintained in CD44KO models. For this reason, we hypothesize that Lrig1+ stem cells probably compensate for the reduced Wnt activity, as Lrig1+ stem cells are still present in the CD44KO mice. This was further supported by cell lineage tracing experiments showing that Lrig1-lineage cells continued to feed the cornea, with however a possible little delay when compared with the wild-type animals (Figure 2).

Together, our findings demonstrate that, as in epidermis, two kinds of stem cells seem to operate in the cornea: the Lrig1-derived and the Wnt activity/CD44-dependent stem cells. The Lrig1-derived cells, by analogy with the epidermis, may rather constitute a reservoir of quiescent stem cells, which play an important role in regenerating the cornea, in case of injury, and in homeostasis. Moreover, the Wnt activity/CD44-dependent stem cells are another cell type that may renew the cornea in homeostatic conditions. In aged corneas, the loss of CD44 expression may induce a reduced Wnt activity with an increased dependency on Lrig1+ stem cells for renewal. Our experiments in mice showed that Lrig1+ stem cells permanently renew the cornea, which is not affected by CD44 loss.

Conversely, suppression of Lrig1+ stem cells in the in vivo ablation experiments also shows that the cornea is able to self-renew without Lrig1+ stem cells, indicating that CD44-dependent cells or other progenitor cells can also sustain epithelial regeneration. This suggests a flexible regenerative system in which Wnt signaling in the basal layer of the cornea continues to support corneal homeostasis. It is important to mention that our study relies on murine models, which may not fully capture the complexity of human corneal biology. Further studies should be performed in order to investigate other epithelial cell types, which may be present in the cornea under various conditions, as well as the interactions between already known markers and stem cells with Lrig1+ stem cells and CD44-dependent stem cells.

In conclusion, our findings indicate two stem cell populations in the corneal epithelium—Lrig1-derived and Wnt/CD44-dependent stem cells—and offer new perspectives on corneal stem cell compartmentalization during aging. Lrig1 has been previously described as a marker of quiescent stem cells in various epithelial tissues, including the skin and the eye, where it contributes to tissue maintenance and injury response [19,20]. On the other hand, CD44 is involved in epidermal homeostasis and Wnt pathway modulation [3]. Our results confirm and extend these observations by showing that CD44-dependent stem cells are active under homeostatic conditions and are associated with Wnt signaling, while Lrig1+ stem cells serve as a quiescent reservoir, predominantly located in the limbus, but they are becoming the primary source of corneal regeneration in aged or CD44-deficient tissues. Additionally, we proved that there is a balance between active and reserve stem cell populations, and this ensures tissue integrity and adaptability.

## 4. Methods

For the human studies, a group of young (5 years old, n = 20) versus aged (>60 years old, n = 20) ocular biopsies were used, and immunofluorescence (IF) was performed to detect the presence of CD44 and Lrig1+ stem cells. Human histological slides were prepared from formalin-fixed paraffin-embedded sections. Slides were treated with citric acid (10 mM, pH 6) for antigen retrieval. Antibodies used: anti-Lrig1 (human) (courtesy of Dr. Satoshi Itami, Osaka University, Osaka, Japan) and anti-CD44 (human) (Bender MedSystems, Vienna, Austria).

A transgenic mouse model was developed in order to perform a topical tamoxifen inducible diphtheria toxin-mediated ablation of Lrig1+ stem cells in the cornea. Transgenic B6.129S6(Cg)Lrig1tm1.1(cre/ERT2)*R26DTA/TOM mice (bearing the Lrig1::CreERT2 and R26::DTA transgenes; Jackson Laboratory, Bar Harbor, ME, USA) and non-transgenic control mice were used in this study. In more detail, the mice that were used in the current study were heterozygotes with a wild-type gene and another one with the Cre-ER (tamoxifen-dependent recombinase) inserted in the Lrig1 exon, right in front of the Lrig1 promoter (small arrow). As for the Rosa26 locus, it had a constitutively activated promoter. Downstream of this promoter, some animals had either the Tomato (TOM) transgene inserted, or DTA (Diptheria toxin fragment A). The expression of both genes was prevented by a stop codon flanked by 2 lox-sequences (target of the CRE). After the application of the 4-hydroxytamoxifen (4-OHT) (Sigma, Saint Louis, MI, USA) (arrow), the 4-OHT bound to the Cre (which is expressed only in cells expressing Lrig1) and so translocated to the nucleus. Once in the nucleus, the Cre removed the stop codon of Tomato/DTA (dashed arrows and ×), so these proteins could be expressed. Cells then became red and started expressing the DTA. The latter was toxic and induced the apoptosis of the given cell (Figure 1).

By using the abovementioned conditional gene modification technology [21], we induced a selective suppression of epidermal cells expressing the Lrig1 gene in the adult mice bearing the Lrig1::CreERT2 and R26::DTA transgenes following topical application of 5μΜ 4-OHT dissolved in ethanol for 14 days (a total of 10 applications). Immunofluorescence was performed in order to detect the presence of Tomato+ cells in samples from control mice and from mice in which Lrig1+ cells were eliminated.

The activation of Wnt signaling pathway, was investigated in WT versus CD44KO mice, by staining the normal beta-catenin (D10A8 XP, rabbit monoclonal antibody, Cell Signaling, Danvers, MA, USA) and the activated form (non-phosphorylated form) of beta-catenin (D13A1, rabbit monoclonal antibody, Cell Signaling, Danvers, MA, USA) in the cornea of WT and CD44KO mice. Finally, cell lineage tracing experiments were performed to detect the role of Lrig1-lineage cells in the samples of WT and CD44KO mice. For these experiments, mice were injected with tamoxifen and corneas were recovered after the sacrifice of the animals at 24 h and 3, 7, and 14 days after tamoxifen injection (Figure 2).

## Data Availability

The original contributions presented in this study are included in the article. Further inquiries can be directed to the corresponding author.

## References

[1] Kaya G., Rodriguez I., Jorcano J.L., Vassalli P., Stamenkovic I. (1997). Selective suppression of CD44 in keratinocytes of mice bearing an antisense CD44 transgene driven by a tissue-specific promoter disrupts. hyaluronate metabolism in the skin and impairs keratinocyte proliferation. Genes Dev..

[2] Kaya G., Saurat J.H. (2007). Dermatoporosis: A chronic cutaneous insufficiency/fragility syndrome-Clinicopathological features, mechanisms, prevention and potential treatments. Dermatology.

[3] Barnes L., Saurat J.H., Kaya G. (2017). Senescent atrophic epidermis retains Lrig1+stem cells and loses Wnt signaling, a phenotype shared with CD44KO Mice. PLoS ONE.

[4] Barnes L., Puenchera J., Saurat J.H., Kaya G. (2015). Lrig1 and CD44v3 Expression in the Human Folliculosebaceous Unit. Dermatology.

[5] Ji Y., Kumar R., Gokhale A., Chao H.P., Rycaj K., Chen X., Li Q., Tang D.G. (2022). LRIG1, a regulator of stem cell quiescence and a pleiotropic feedback tumor suppressor. Semin. Cancer Biol..

[6] Jensen K.B., Collins C.A., Nascimento E., Tan D.W., Frye M., Itami S., Watt F.M. (2009). Lrig1 Expression Defines a Distinct Multipotent Stem Cell Population in Mammalian Epidermis. Cell Stem Cell.

[7] Clayton E., Doupé D.P., Klein A.M., Winton D.J., Simons B.D., Jones P.H. (2007). A single type of progenitor cell maintains normal epidermis. Nature.

[8] Lim X., Tan S.H., Koh W.L.C., Chau R.M.W., Yan K.S., Kuo C.J., Van Amerongen R., Klein A.M., Nusse R. (2013). Interfollicular epidermal stem cells self-renew via autocrine Wnt signaling. Science.

[9] Page M.E., Lombard P., Ng F., Göttgens B., Jensen K.B. (2013). The epidermis comprises autonomous compartments maintained by distinct stem cell populations. Cell Stem Cell.

[10] Jensen K.B., Watt F.M. (2006). Single-cell expression profiling of human epidermal stem and transit-amplifying cells: Lrig1 is a regulator of stem cell quiescence. Proc. Natl. Acad. Sci. USA.

[11] Nakamura T., Hamuro J., Takaishi M., Simmons S., Maruyama K., Zaffalon A., Bentley A.J., Kawasaki S., Nagata-Takaoka M., Fullwood N.J. (2014). LRIG1 inhibits STAT3-dependent inflammation to maintain corneal homeostasis. J. Clin. Investig..

[12] Masterton S., Ahearne M. (2018). Mechanobiology of the corneal epithelium. Exp. Eye Res..

[13] Nurković J.S., Vojinović R., Dolićanin Z. (2020). Corneal Stem Cells as a Source of Regenerative Cell-Based Therapy. Stem Cells Int..

[14] Ruan Y., Jiang S., Musayeva A., Pfeiffer N., Gericke A. (2021). Corneal epithelial stem cells-physiology, pathophysiology and therapeutic options. Cells.

[15] Li J., Xiao Y., Coursey T.G., Chen X., Deng R., Lu F., Pflugfelder S.C., Li D.Q. (2017). Identification for Differential Localization of Putative Corneal Epithelial Stem Cells in Mouse and Human. Sci. Rep..

[16] Davidson K.C., Sung M., Brown K.D., Contet J., Belluschi S., Hamel R., Moreno-Moral A., dos Santos R.L., Gough J., Polo J.M. (2024). Single nuclei transcriptomics of the in situ human limbal stem cell niche. Sci. Rep..

[17] Cotsarelis G., Cheng S.-Z., Dong G., Sun T.-T., Lavker’ R.M. (1989). Existence of Slow-Cycling Limbal Epithelial Basal Cells That Can Be Preferentially Stimulated to Proliferate: Implications on Epithelial Stem Cells. Cell.

[18] Majo F., Rochat A., Nicolas M., Jaoudé G.A., Barrandon Y. (2008). Oligopotent stem cells are distributed throughout the mammalian ocular surface. Nature.

[19] Watt F.M., Jensen K.B. (2009). Epidermal stem cell diversity and quiescence. EMBO Mol. Med..

[20] Yang W., Lee S.K., Lehmann O.J., Wu Z., Hiriyanna S., Swaroop A., Lavker R.M., Peng H., Kume T. (2023). FoxC1 activates limbal epithelial stem cells following corneal epithelial debridement. Exp. Eye Res..

[21] Sharma S., Zhu J. (2014). Immunologic applications of conditional gene modification technology in the mouse. Curr. Protoc. Immunol..

